# Evaluation of a stent dressing and abdominal bandage on surgical site infection following emergency equine laparotomy: A randomised controlled trial

**DOI:** 10.1111/evj.14482

**Published:** 2025-02-19

**Authors:** Cajsa M. Isgren, Gina L. Pinchbeck, Shebl E. Salem, Michelle J. Hann, Neil B. Townsend, Matthew D. Cullen, Debra C. Archer

**Affiliations:** ^1^ School of Veterinary Science, Institute of Infection, Veterinary & Ecological Sciences University of Liverpool, Leahurst Campus Neston Wirral UK; ^2^ Department of Surgery, Anaesthesiology, and Radiology, Faculty of Veterinary Medicine Zagazig University Zagazig Egypt

**Keywords:** horse, laparotomy, randomised controlled trial, stent dressing, surgical site infection

## Abstract

**Background:**

Surgical site infection (SSI) is a frequent complication following emergency equine laparotomy, negatively impacting equine welfare, increasing treatment costs and presenting a hospital biosecurity risk.

**Objectives:**

To determine if a sutured‐on stent dressing for incisional protection during anaesthetic recovery reduced SSI following emergency laparotomy.

**Study Design:**

Randomised controlled trial.

**Methods:**

Eligible horses were randomised to a sutured‐on stent (intervention) or textile dressing (control) as the primary component of a 3‐layer abdominal bandage placed for anaesthetic recovery. Horses were followed up to 90 days postoperatively. Data were analysed according to intention‐to‐treat principles. Time to SSI (primary outcome) for each group was analysed using a Cox proportional hazard model. Secondary outcomes (SSI and pyrexia during hospitalisation, days hospitalisation and incisional hernia formation at 90 days) were analysed using Chi‐squared tests and a univariable logistic regression model (categorical data) or by comparing means between groups (continuous data).

**Results:**

Of 352 eligible horses enrolled (167 intervention group, 185 control group), SSI developed in 101 (28.7%) at a mean of 9.7 days (SD 4.6 days). Rate of SSI was not significantly different between groups unadjusted (hazard ratio [HR] 0.83, 95% CI 0.56–1.23, *p* = 0.4) or adjusted for variables significantly associated with rate of SSI (HR 0.88, 95% CI 0.59–1.30, *p* = 0.5). There were no significant differences in secondary outcomes between intervention and control groups.

**Main Limitations:**

Single‐centre study evaluating incisional protection from a primary dressing under a secondary adhesive and tertiary fabric abdominal bandage for anaesthetic recovery.

**Conclusions:**

Use of a sutured‐on stent compared with a textile adhesive dressing as the primary layer of an abdominal bandage for anaesthetic recovery did not reduce the rate of SSI. Further RCT are warranted to investigate efficacy of other interventions on reduction of SSI following emergency laparotomy in horses.

## 1 INTRODUCTION

Surgical site infection (SSI) is one of the most frequent complications following emergency exploratory laparotomy (celiotomy), usually undertaken to treat equine colic, with a reported prevalence of 7.4%–43%.^1^, ^2^ SSI is important from an equine welfare and economic perspective due to prolonged postoperative pain, delayed incisional healing, additional treatment required, increased duration of hospitalisation and associated additional costs. SSI also increases the risk of incisional dehiscence and abdominal hernia formation, horses that develop the latter complication being 7–14 times less likely to return to athletic function.^3^, ^4^ Prolonged or additional antimicrobial treatment also increases the risk of antimicrobial resistance (AMR) and isolation of multidrug resistant (MDR) bacteria from SSI is an equine hospital biosecurity and public health risk.^5^ Therefore, strategies to minimise the likelihood of SSI following emergency laparotomy in horses are important.

Multiple risk factors for SSI following emergency equine laparotomy have been reported with inconsistent and sometimes conflicting findings reported between different studies.^1^, ^2^ Identified risk factors for SSI that are modifiable such as method of incisional closure,^6^ application of medical grade honey gel during incisional closure^7^ or method of postoperative incisional protection^8^, ^9^ have been the focus of a small number of randomised controlled trials (RCTs). Minimising bacterial contamination of the incision intraoperatively and during the early postoperative period, particularly during anaesthetic recovery, has been proposed to reduce the risk of SSI in horses.^10^, ^11^, ^12^ However, there is limited high‐quality evidence of efficacy of different types of incisional protection to reduce SSI from appropriately designed and suitably powered RCTs and methods currently vary widely between equine clinics. One RCT^8^ demonstrated that application of an abdominal bandage in the postoperative period following exploratory laparotomy significantly reduced incisional complications, but there is currently limited information based on sufficiently powered RCT regarding methods of incisional protection during anaesthetic recovery.

Physical protection of an abdominal incision during equine anaesthetic recovery can be challenging due to patient size, physical forces placed on the incision and incisional dressings and lack of adherence of adhesive dressings that are designed for human skin. Our hospital protocol for protection of ventral midline laparotomy incisions following emergency laparotomy consists of a three‐layer abdominal bandage placed for anaesthetic recovery which is removed once the horse is moved from the anaesthetic recovery box (Methods S1). All layers are then removed and a new abdominal bandage placed, based on evidence from an RCT.^8^ However, adherence of the primary dressing and adhesive drape to the skin immediately following closure of the incision was observed to be variable between horses and prone to becoming dislodged, sometimes exposing part or all of the incision during anaesthetic recovery. Publication of study demonstrating a protective effect of a sutured‐on stent dressing on SSI prevalence^13^ and concurrent launch of a commercial equine laparotomy stent dressing (Equine Stent bandage, Kruuse) was a stimulus for review of our protocol for incisional protection during anaesthetic recovery to minimise development of SSI.

Sutured‐on stent dressings, originally demonstrated to provide incisional protection, absorption of tissue fluid and reduced risk of infection in people^14^ have been proposed to improve physical protection of equine laparotomy incisions.^13^ These are stated in the equine literature as a method to potentially reduce SSI prevalence, but critical appraisal of the available published evidence at the time of protocol review for the present study revealed limited and conflicting evidence of efficacy. One study reported increased frequency of SSI associated with use of a sutured‐on stent bandage,^11^ two studies reported reduced frequency of SSI^13^, ^15^ and two studies reported no apparent effect.^16^, ^17^ However, the latter studies were retrospective and non‐randomised and therefore subject to a number of potential biases. A randomised study by Kilcoyne et al.^9^ has since been published that reported a protective effect of sutured‐on stent dressings. Taking an evidence‐based veterinary medicine (EBVM) approach^18^ we elected to undertake an RCT to determine if incorporation of a sutured‐on stent dressing into our hospital protocol for incisional protection during anaesthetic recovery had any effect on reduction of postoperative SSI in our equine patients.

The primary objective of this RCT was to assess the efficacy of a commercial, sutured‐on stent dressing (intervention) compared with a standard adhesive dressing (control) as the primary layer of a 3‐layer abdominal bandage placed for anaesthetic recovery on rates of SSI following equine emergency laparotomy. Secondary objectives were to evaluate differences in SSI prevalence and pyrexia during hospitalisation, duration of hospitalisation and incisional hernia formation at 90 days between intervention and control groups.

## 2 MATERIALS AND METHODS

### 2.1 Trial design and patients

**FIGURE 1:**
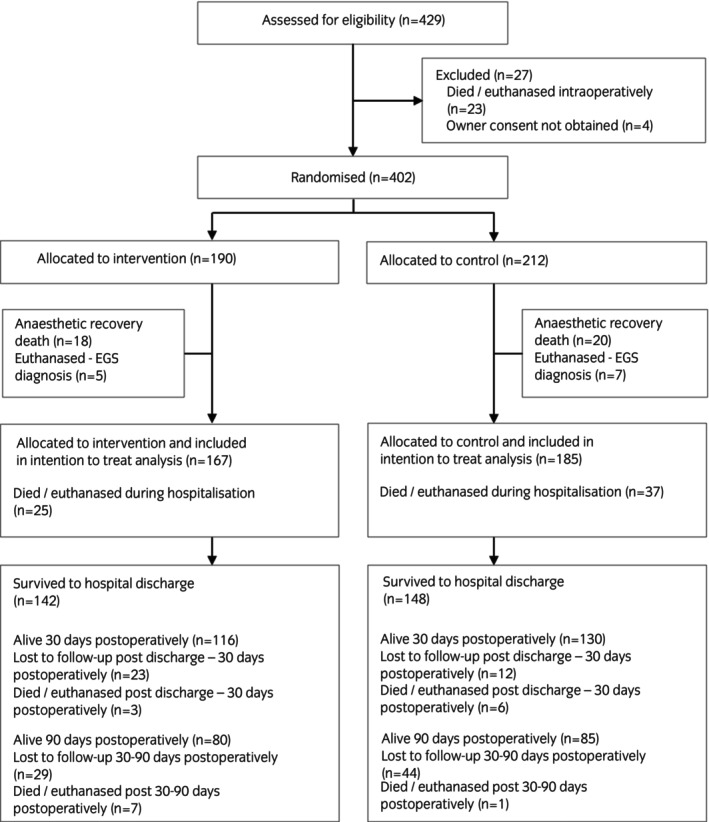
CONSORT diagram showing the flow of participants in each stage of the trial. EGS, equine grass sickness.

This was a parallel group, RCT. Protocol design (Protocol S1) and study reporting were conducted in accordance with CONSORT 2010 guidelines.^19^ All horses >1 year of age undergoing emergency exploratory laparotomy under general anaesthesia at a single equine referral hospital (Philip Leverhulme Equine Hospital, University of Liverpool) were eligible for enrolment. Horses that died or were euthanased during surgery or anaesthetic recovery, where informed owner consent was not obtained or which were euthanased following confirmation of a diagnosis of acute equine grass sickness (EGS), based on ileal biopsy results, were excluded (Figure 1).

Hospital audit data indicated that SSI were expected to develop in approximately 20% of horses following emergency laparotomy. Based on a reduction in SSI in the intervention (stent) group to 10% which was considered to be clinically meaningful, to have 80% power to detect a difference between groups with 95% confidence, a sample size of 197 horses in each group (394 horses total) was required.^20^ SSI was defined as any purulent discharge from the laparotomy incision or more than 24 h of serous discharge with at least one of the following clinical signs: pain, localised swelling, erythema or heat.^10^

Pre‐, intra‐ and postoperative data were recorded contemporaneously on standard hospital recording forms. Preoperative data recorded included patient age, sex, breed, weight, heart rate, packed cell volume and peripheral lactate at hospital admission. Coat condition prior to surgical preparation, date and time of surgery (out‐of‐hours [OOH] defined as surgery performed between the hours of 5 PM and 9 AM Monday–Friday and at any time over the days of Saturday or Sunday^21^), lesion type and location, and surgical procedures performed were also recorded. During abdominal closure, incisional length, and retroperitoneal fat depth (cm) were measured. Surgeon, duration of surgery, duration of general anaesthesia and anaesthetic recovery score^22^ were also recorded and postoperative progress including date of hospital discharge were documented on standard hospital recording forms. Post‐operative pyrexia was defined as a rectal temperature >38.6°C on at least one occasion^10^ during the period of hospitalisation. Adverse events including acute abdominal dehiscence (AAD), repeat laparotomy (RL) or euthanasia/death were also recorded.

Perioperative antimicrobial (penicillin/gentamicin) and analgesic therapy were administered according to standard hospital practice, modified according to patient status, response to therapy and attending clinician preference. The 3‐layer abdominal bandage, including the allocated primary dressing, was removed immediately following anaesthetic recovery and was replaced with a new abdominal dressing and bandage (Methods S1). Abdominal bandages and dressings were changed every 48 h, sooner if there was evidence of exudate through the dressing. The incision was inspected by the veterinary clinician(s) responsible for the care of each case at the time of bandage changes and SSI was recorded as being present or absent. The bandage was maintained in place for at least two dressing changes and was discontinued according to clinical progress and clinician judgement. Where SSI developed, incisional discharge was sampled aseptically using a charcoal medium transport swab (Deltalab) following cleaning of the incision using 0.1% povidone iodine and was submitted to the diagnostic veterinary microbiology laboratory at the University of Liverpool for culture and susceptibility testing (see Table S1 for details and microbiology results).

Data for each patient were recorded on a specifically designed spreadsheet (Microsoft Excel) and were cross‐checked with theatre allocation and hospital records where data omissions or inconsistencies were identified.

### 2.2 Randomisation and blinding

Simple random allocation (allowing unequal group sizes) was used with a 1:1 ratio to either intervention or control group using a concealed, envelope‐based system. A random number generator (Microsoft Excel) was used to generate a randomised allocation sequence. Individual opaque envelopes were numbered sequentially and the allocation of incisional protection for that numbered envelope to the intervention (sutured‐on stent) or control (standard adhesive textile dressing) group was placed inside, each envelope was sealed, and these were stored in numeric order in the operating theatre. At the end of laparotomy, closure of the midline incision was performed in the same way by most surgeons using 5 metric braided lactomer loop (double layer) in a simple continuous pattern to close the lina alba followed by closure of the skin using 3.5 metric polypropylene in a ford interlocking or continuous suture pattern. Modifications included a single suture of 5 metric braided lactomer (central cruciate interrupted suture and continuous pattern on either side) and an additional subcutaneous layer (3 metric braided lactomer or other absorbable suture) based on surgeon preference. Lavage of the incision using sterile lactated ringers or saline solution was performed in some cases based on surgeon preference and surgical procedures undertaken. No antimicrobial agents were applied to the incision during closure. Once patient eligibility and inclusion/exclusion criteria had been confirmed (see Protocol S1), randomisation was performed. Theatre personnel opened the next envelope in the numeric sequence, the allocated method of incisional protection for anaesthetic recovery was stated to the surgical team and was recorded on a theatre‐based paper spreadsheet. Blinding was not practical due to limitations in hospital personnel available to perform confirmed blinded assessment of incisions postoperatively. All incision assessors were experienced veterinary surgeons and were provided with the standardised criteria for SSI. Allocation of horse to intervention or control group was not detailed in the patient records to minimise any potential bias when postoperative evaluation of the incision was performed nor were horse owners or each horse's usual treating veterinary surgeon aware of allocation group.

### 2.3 Intervention

The intervention and control groups were discussed between the investigators who designed and set up the study (CMI, NBT, DCA, GP) with feedback from the wider hospital team prior to the protocol being finalised. Application of the sutured‐on stent dressing as the sole form of incisional protection raised concerns within the team around potential to become saturated with urine or blood during anaesthetic recovery and consequent incisional bacterial contamination. The same type of water‐impervious protective adhesive drape used to cover the primary dressing in our existing protocol had also been applied over the stent dressing for anaesthetic recovery in the Tnibar et al. study.^13^ In the latter study after removal of the drape, the stent was then left in place for up to 5 days which is different to the present study. Application of the adhesive textile dressing and drape without the fabric elasticated bandage, used to provide some external support and protection of the surgical site during anaesthetic recovery, also raised concerns around increased potential for the adhesive dressing and drape to become dislodged and the incision to become exposed. As we wished to compare only the two types of primary dressings (and not the additional effect of the fabric elasticated abdominal bandage), consensus was achieved by choosing to evaluate the sutured‐on stent dressing versus the current adhesive textile dressing as the first layer of the standard 3‐layer abdominal bandage for anaesthetic recovery. Based on RCT evidence of reduction of SSI using our existing method of incisional protection placed after anaesthetic recovery,^8^ we chose to focus only on evaluation of the two different methods of incisional protection for anaesthetic recovery.

For horses allocated to the intervention group, once skin closure had been completed, a commercial stent dressing (Equine Stent Bandage, Kruuse) was sutured to the skin to cover the incision using six sutures of 4 metric polypropylene. In the control group, following skin closure, a sterile conformable textile dressing with an adhesive backing (Primapore, Smith & Nephew) was applied over the incision. In both groups, this was followed by placement of an adhesive dressing (Opsite, Smith & Nephew) over the dressing and application of an elasticated fabric abdominal bandage (OrthoHorse, VetExtras) as the horse was hoisted off the operating table and was moved into the anaesthetic recovery box. Immediately following anaesthetic recovery and once the horse was standing and ready to be moved from the recovery box, the abdominal bandage including the allocated primary dressing were removed and a standard (hospital protocol) abdominal bandage was immediately applied to all horses. This consisted of a sterile absorptive primary contact layer (Melonin, Smith & Nephew) placed over the incision held in place with an elasticated cohesive dressing (Vet‐Flex, Kruuse) wrapped around the abdomen and secured using an elastic adhesive bandage (Tensoplast, BSN medical), as shown in Methods S1.

### 2.4 Efficacy outcomes

The primary outcome was time (days) to development of SSI. Secondary outcomes were proportion of horses that developed a SSI and pyrexia during hospitalisation, duration of hospitalisation and incisional hernia formation at 90 days postoperatively. Long term follow‐up data were collected via a standardised telephone questionnaire with owners (see Questionnaire S1). Where owners could not be contacted, postoperative follow‐up data for the time period of interest were obtained from the horse's usual veterinary practice.

### 2.5 Data analysis

Data were analysed according to intention to treat principles (ITT) using Stata (Stata Statistical Software Release 18, StataCorp LLC). Baseline data for each group (intervention or control) were assessed and continuous variables reported as means and standard deviation (Normally distributed data) or as median and interquartile range (IQR) for non‐Normally distributed data. Baseline categorical data were assessed and reported as numbers and proportions. The primary outcome (days to SSI) was analysed using time‐to‐event analysis. Horses were censored at the point of death/euthanasia or at time of loss to follow up. Time to SSI for each group was estimated according to the Kaplan–Meier method and was analysed using a Cox proportional hazard model. Variables considered a priori to potentially alter the likelihood of SSI were evaluated including RL, and the effect of surgeon was assessed as a random effect in the model. Variables significantly associated with SSI were included in a final model to provide an adjusted HR according to intervention or control group. For secondary outcomes, categorical data were analysed using Chi‐squared tests and a univariable logistic regression model. Continuous data were analysed by comparing means between groups (*t*‐test). Statistical significance was set as *p* < 0.05.

## 3 RESULTS

### 3.1 Study participants

Between March 2014 and July 2018, 429 horses were assessed for trial eligibility and 402 were enrolled onto the trial (Figure 1). A total of 190 horses were randomised to the intervention group and 212 to the control group. Of these, a total of 352 horses (167 horses in the intervention group and 185 horses in the control group) were included in the study and were analysed using ITT. Patient signalment and presenting features (Table 1) and operative features (Table 2) did not differ substantially between the two groups at baseline.

**TABLE 1:** Patient and presenting features in horses (*n* = 352) undergoing emergency laparotomy in which the incision was protected for anaesthetic recovery with a stent dressing (intervention) or standard adhesive dressing (control).

Patient/presenting features	Intervention (*n* = 167)	Control (*n* = 185)
Age (years), mean (SD)	11.9 (6.2); *n* = 167, (100%)	12.2 (6.1); *n* = 185, (100%)
Weight (kg), mean (SD)	527.0 (130.8); *n* = 166, (99.4%)	519.4 (130.6); *n* = 185, (100%)
Heart rate at admission (bpm), mean (SD)	52.3 (16.4); *n* = 164, (98.2%)	55.5 (15.8); *n* = 182, (98.4%)
Packed cell volume at admission (%), mean (SD)	37.9 (8.0); *n* = 164, (98.2%)	39.1 (7.7); *n* = 182, (98.4%)
Systemic lactate at admission (mmol/L), median (IQR)	1.40 (0.50–2.47); *n* = 155, (92.8%)	1.32 (0.50–2.20); *n* = 176, (95.1%)
Sex number (%)		
Female	69 (41.3)	62 (33.5)
Male	98 (58.7)	123 (66.5)
Breed number (%)		
Thoroughbred/Thoroughbred x	34 (20.3)	42 (22.7)
Other light horse breed	61 (36.6)	53 (28.7)
Pony/Cob/Donkey	51 (30.5)	70 (37.8)
Draught horse	21 (12.6)	20 (10.8)
Coat condition at admission number (%)		
Clean unclipped	76 (45.5)	85 (46.0)
Clean clipped	22 (13.2)	30 (16.2)
Moderate	28 (16.8)	37 (20.0)
Filthy	20 (12.0)	15 (8.1)
Not recorded	21 (12.6)	18 (9.7)
Season of admission number (%)		
March–May (Spring)	52 (31.1)	58 (31.3)
June–August (Summer)	50 (30.0)	51 (27.6)
September–November (Autumn)	33 (19.7)	33 (17.9)
December–February (Winter)	32 (19.2)	43 (23.2)

Abbreviations: IQR, interquartile range; SD, standard deviation.

**TABLE 2:** Perioperative features in horses (*n* = 352) undergoing emergency laparotomy in which the incision was protected for anaesthetic recovery with a stent dressing (intervention) or standard adhesive dressing (control).

Perioperative features	Intervention (*n* = 167)	Control (*n* = 185)
Incision length (cm), mean (SD)	23.4 (4.5); *n* = 161, (96.4%)	22.7 (4.2); *n* = 172, (93.0%)
Retroperitoneal fat depth (cm), mean (SD)	2.4 (1.3); *n* = 160, (95.8%)	2.6 (1.3); *n* = 175, (95.6%)
Surgery time (min), mean (SD)	106.5 (42.8); *n* = 167, (100%)	106.2 (45.1); *n* = 183, (98.9%)
Anaesthetic duration (min), mean (SD)	134.3 (44.6); *n* = 167, (100%)	134.1 (46.7); *n* = 183, (98.9%)
Primary lesion type number (%)		
Small intestinal non‐strangulating	29 (17.4)	29 (15.7)
Small intestinal strangulating	62 (37.1)	65 (35.1)
Large colon non‐strangulating	49 (29.3)	57 (30.8)
Large colon strangulating	9 (5.4)	16 (8.7)
Other	18 (10.8)	18 (9.7)
Surgery performed OOH number (%)		
Yes	107 (64.1)	123 (66.5)
No	60 (35.9)	62 (33.5)
Enterotomy performed number (%)		
Yes	81 (48.5)	85 (45.9)
No	85 (50.9)	100 (54.0)
Not recorded	1 (0.6)	‐
Pelvic flexure enterotomy number (%)		
Yes	72 (43.1)	76 (41.1)
No	94 (56.3)	109 (58.9)
Not recorded	1 (0.6)	‐
Intestinal resection number (%)		
Yes	44 (26.3)	49 (26.5)
No	122 (73.1)	136 (73.5)
Not recorded	1 (0.6)	‐
Typhlotomy number (%)		
Yes	18 (10.8)	14 (7.6)
No	148 (88.6)	171 (92.4)
Not recorded	1 (0.6)	‐
Incisional lavage number (%)		
Yes	88 (52.7)	98 (53.0)
No	41 (24.5)	57 (30.8)
Not recorded	38 (22.8)	30 (16.2)
Linea alba closure number (%)		
Single loop	145 (86.8)	156 (84.3)
Other	16 (9.6)	25 (13.5)
Not recorded	6 (3.6)	4 (2.2)
Incision closure layers number (%)		
Three	161 (96.4)	179 (96.7)
Two	1 (0.6)	2 (1.1)
Not recorded	5 (3.0)	4 (2.2)
Suture pattern in skin number (%)		
Ford interlocking	117 (70.1)	127 (68.7)
Simple continuous	43 (25.7)	53 (28.6)
Other/Not recorded	7 (4.2)	5 (2.7)
Anaesthetic recovery score number (%)		
1 (Excellent)	25 (15.0)	23 (12.4)
2 (Good)	60 (35.9)	65 (35.1)
3 (Fair)	64 (38.3)	58 (31.4)
4 (Marginal)	5 (3.0)	14 (7.6)
5 (Poor/unsuccessful)	1 (0.6)	1 (0.5)
Not recorded	12 (7.2)	24 (13.0)
Incisional protection once stood number (%)		
No exposure of incision	38 (22.7)	27 (14.6)
Partial exposure of incision	7 (4.2)	6 (3.2)
Complete exposure of incision	13 (7.8)	21 (11.4)
Not assessed	109 (65.3)	131 (70.8)
Antimicrobial surgical prophylaxis number (%)		
Penicillin	24 (14.4)	30 (16.2)
Penicillin and gentamicin	138 (82.6)	150 (81.1)
Cephalosporin	4 (2.4)	1 (0.5)
Not recorded	1 (0.6)	4 (2.2)
Duration of all antimicrobial therapy (days), mean (SD)	5.6 (2.4); *n* = 166, (99.4%)	5.1 (2.1); *n* = 181, (97.8%)
Postoperative colic number (%)		
Yes	42 (25.1)	59 (32.4)
No	123 (73.7)	123 (67.6)
Not recorded	2 (1.2)	‐
Relaparotomy number (%)		
Yes	10 (6.0)	26 (14.1)
No	157 (94.0)	159 (85.9)

Abbreviation: SD, standard deviation.

### 3.2 Surgical site infection

**FIGURE 2:**
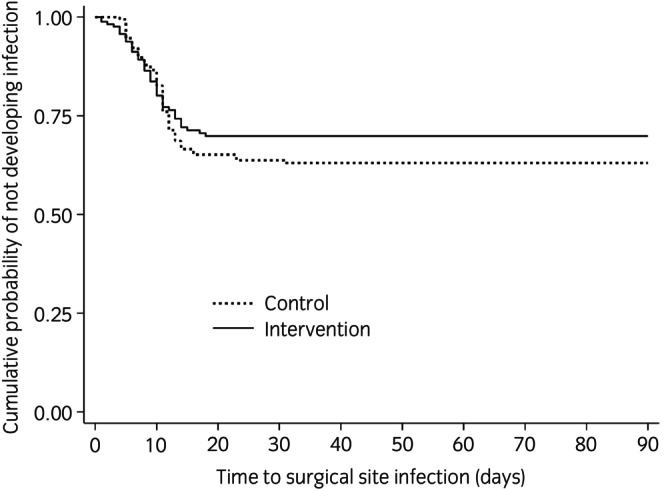
Kaplan–Meier curves for Control and Intervention groups comparing the rate of surgical site infection (SSI) between groups in the 90‐day follow up period. A Cox proportional hazards model demonstrated this difference between groups to be non‐significant (*p* = 0.4).

A total of 101 horses (28.7%) developed a SSI at a mean of 9.7 days (SD 4.6 days) postoperatively (Table 3). There was no significant difference in the rate of SSI between the intervention and control groups when evaluated using Kaplan–Meier curves (Figure 2) and in a univariable Cox proportional hazard model (HR 0.83, 95% CI 0.56–1.23, *p* = 0.4; Table 3). This did not change when surgeon (*n* = 11) was included as a random effect in the model. When other variables were tested in the model only RL (HR 3.1, 95% CI 1.74–5.52, *p* < 0.001) and typhlotomy (HR 1.95, 95% CI 1.12–3.37, *p* = 0.02) were significantly associated with time to SSI. In a Cox multivariable proportional hazards regression model fitted with the variables RL, typhlotomy and incisional protection for anaesthetic recovery (intervention/control), there remained no significant difference in rate of SSI between intervention and control groups (adjusted HR 0.88, 95% CI 0.59–1.30, *p* = 0.5).

**TABLE 3:** Primary and secondary outcomes in 352 horses undergoing emergency laparotomy in which the ventral midline incision was protected during anaesthetic recovery with a stent (intervention) or standard adhesive dressing (control).

Outcome	Intervention	Control	Estimate (95% CI)	*p* Value
Primary outcome				
Surgical site infection (SSI)	44/167 (26.4%)	57/185 (30.8%)	HR 0.83 (0.56–1.23)	0.4
Secondary outcomes				
SSI during hospitalisation	30/167 (18.0%)	36/185 (19.5%)	OR 0.91 (0.53–1.55)	0.7
Pyrexia during hospitalisation	72/167 (43.1%)	94/185 (50.8%)	OR 0.71 (0.46–1.08)	0.1
Days hospitalisation, mean (SD)	10.0 (4.9)	9.7 (5.5)	0.33^a^ (−1.43–0.77)	0.6
Incisional hernia at 90 days	10/167 (6.0%)	8/185 (4.3%)	OR 1.32 (0.86–2.03)	0.2

Abbreviations: HR, hazard ratio (unadjusted); OR, odds ratio (unadjusted).

^a^ Difference in means.

### 3.3 Secondary outcomes

SSI during hospitalisation occurred in 66 horses (18.8%) and was not significantly different between intervention and control groups (Table 3). Results of culture and susceptibility testing for those horses are shown in Table S1. There was no substantial difference in the proportion of horses that developed Gram‐positive or Gram‐negative infections between groups. In horses that developed SSI (*n* = 101) just over a third of these (34.6%) were identified following hospital discharge up to a maximum of 31 days postoperatively. A total of 166 horses (47.2%) developed pyrexia during hospitalisation and again, this was not significantly different between groups. Pyrexia was significantly associated with SSI (*p* = 0.003) which developed in 61 of these horses (36.7%). Neither duration of hospitalisation (mean 9.87 days, SD 5.2) nor incisional hernia formation by 90 days postoperatively (*n* = 18; 5.1%) differed significantly between groups (Table 3).

### 3.4 Repeat laparotomy

A total of 36 horses (10.2%) underwent RL at a median of 4 days (IQR 2.5–7.5 days) following initial laparotomy (Table 2). RL was performed during the same period of hospitalisation in all but two horses (25 and 51 days following initial laparotomy and subsequent hospital discharge). Fourteen horses (38.9%) were euthanased during RL (intervention group *n* = 4, 40.0%; control group *n* = 10, 38.4%). SSI developed in a total of 16 horses (44.4%) that underwent RL. Sensitivity analysis was performed by censoring horses at the time of RL (in addition to time of death or loss to follow‐up) for the primary outcome of interest (time to SSI). The effect of intervention or control dressing on time to SSI remained non‐significant (HR 0.91, 95% CI 0.53–1.55, *p* = 0.7).

### 3.5 Adverse events and postoperative survival

No adverse events specifically related to the intervention (sutured‐on stent) were observed. Acute abdominal dehiscence (AAD) occurred in three horses during hospitalisation (0.85%; intervention group *n* = 3) of which one underwent surgical repair and the other two were euthanased. AAD was not considered to be related to use of the intervention in any of these cases. A total of 290 horses (82.4%) included in the study survived to hospital discharge (85.0% in the intervention group, 80.0% in the control group; Figure 1). For those horses and where follow‐up data were available at 30 and 90 days postoperatively, 246 (96.5%) and 165 (90.7%) respectively were confirmed to still be alive.

## 4 DISCUSSION

This RCT found that use of a commercial, sutured‐on‐stent compared with a standard textile adhesive dressing as the primary component of a 3‐layer abdominal bandage for incisional protection during anaesthetic recovery following equine emergency laparotomy did not alter the rate of SSI in our hospital population. This finding did not change when controlled for factors associated with SSI and for horses that developed SSI during and following hospitalisation. In addition, there was no significant difference between either group on proportion of horses that developed pyrexia during hospitalisation, duration of hospitalisation or prevalence of incisional hernia formation. This study provides additional contribution to high‐level evidence provided by RCT, including investigation of optimal methods to prevent SSI following equine emergency laparotomy.

The proportion of horses that developed SSI during hospitalisation (18.8%) in the present study was similar to the results of previous research and clinical audits conducted in our hospital,^10^, ^23^ and was within reported ranges from other equine hospital populations.^1^ Overall, prevalence of SSI was greater (28.7%) due to around a third of all SSIs only developing following hospital discharge and up to 31 days postoperatively. This demonstrates the importance of follow‐up beyond hospital discharge to accurately report SSI following emergency equine laparotomy. The latter is inconsistently performed in some published studies and if undertaken, use of variable criteria for SSI recording and end‐points for measurement of SSI make accurate comparison between studies difficult.^2^ In studies assessing the effect of interventions to reduce SSI in people, SSI by 30 days is a common primary outcome measure used.^24^, ^25^, ^26^ We propose that ‘SSI by 30 days’ is an outcome measured and reported in all studies of SSI following emergency equine laparotomy to provide a more accurate measure of SSI prevalence and facilitating comparisons between studies including formal meta‐analyses.

Subsequent to conclusion of the present RCT, a randomised controlled study that evaluated 3 different types of incisional protection applied following equine emergency laparotomy has been published.^9^ The latter study evaluated the effect of two different types of sutured‐on stent dressings (sterile cotton towel or polyhexamethylene biguanide‐impregnated dressing) and sterile gauze covered by an iodine‐impregnated adhesive drape, placed at the end of incisional closure. Incisional complications were more likely to occur in the adhesive drape group compared with both stent groups. However, the study was small (85 horses in total, *n* = 24, 26 and 25 in groups 1–3 respectively) and subject to potential Type I error, that is, reporting a significant association where this may be a chance finding due to a small sample size that did not account for random variation.^19^, ^27^ Ideally randomised studies should individually have sufficient power,^28^ although publication of smaller, low powered RCT are important making them available for subsequent meta‐analysis.^29^ Additional, RCT that are conducted and reported in accordance with CONSORT guidelines^19^ are therefore justified to evaluate the efficacy of these and other interventions to prevent SSI following emergency laparotomy.

Direct comparison between the findings from the present study and studies evaluating a sutured‐on stent as a sole method of incisional protection during anaesthetic recovery cannot be made. However, this was not the reason for conducting this study, which was to answer a clinical question in our hospital using EBVM principles. Our decision and justification to evaluate a sutured‐on stent dressing for anaesthetic recovery only and as one component of a 3‐layer abdominal bandage is described in the methods section. Consensus from relevant stakeholders during trial design and protocol development is important when setting up RCT and helped to maximise compliance with the study protocol and recruitment of eligible horses by the hospital team in the present study.

Emergency equine laparotomy has a high perioperative mortality rate compared with horses undergoing elective surgical procedures,^30^ and the rate of mortality is greatest within the first 10 days postoperatively.^31^ Despite factoring in an expected drop‐out rate following randomisation, the number of horses with data for analysis using ITT was slightly lower than we aimed to achieve based on original power calculations. It was not possible to randomise additional horses due to discontinuation of the product by the manufacturer towards the end of the study and due to personnel changes. However, the overall prevalence of SSI was greater than the prevalence used in the original power calculations, thereby increasing study power. Time‐to‐event analysis was also used to analyse the primary outcome enabling data from horses that died or were lost to follow‐up to be included, avoiding loss of study power compared with use of a dichotomous (SSI—Yes/No) outcome.^32^ Whilst dichotomous outcomes are often used in human SSI studies, the rate of mortality following emergency equine laparotomy (including euthanasia on welfare or economic grounds) and loss to follow‐up is often greater. Time‐to‐event analysis is infrequently used in analysis of studies assessing the effects of interventions on SSI following equine laparotomy.^6^ More frequent use of a dichotomous outcome not only reduces study power but also exclusion of data from horses that died, were lost to follow up or other sub‐groups has the potential for bias associated with non‐random loss of participants and should be avoided in the primary analysis of RCT.^19^ Time‐to‐event analysis is a more robust statistical method which we consider should be used more consistently in interventional studies investigating SSI following emergency equine laparotomy.

Variables previously shown to alter the risk of SSI following emergency equine laparotomy were incorporated into data measured in the present study to assess for any differences between the intervention and control groups at the point of randomisation that could affect the results and to enable analysis adjusted for effects of key variables to be reported.^19^ There were no substantial differences between these groups confirming that randomisation was effective in the present study. In addition to avoiding biases that may be inherent to retrospective studies, randomisation also enables the effect of any unknown or unmeasured factors to be accounted for. Whilst this RCT did not find any evidence that the intervention being investigated reduced SSI, it also did not find any evidence of any increased risk of SSI. Reporting and publication of RCT that do not report a positive finding are important in order to avoid publication bias.^33^

Horses that undergo RL have a higher prevalence of SSI and it has been identified as a risk factor in previous studies.^11^ In the present study, RL increased the risk of SSI three‐fold. Horses in which RL was undertaken were not excluded from analysis as per ITT principles and to avoid sub‐group analysis being performed for the reasons already stated. The proportion of horses that underwent RL was greater in the control group (14.1%) compared with the intervention group (6.0%) but is unlikely to have any relation to either method of incisional protection. Sensitivity analysis performed by repeating analysis with horses censored at the point of RL confirmed that inclusion of horses that underwent RL had no effect on the study findings.

Horses in which a typhlotomy was performed (*n* = 32) had double the risk of SSI in the present study and was an unexpected finding. Typhlotomy was most commonly performed to relieve caecal distention following decompression of small intestinal fluid content into the caecum, most frequently during management of a primary small intestinal luminal obstruction (*n* = 16, including ileal impaction and idiopathic focal eosinophilic enteritis). Primary caecal dysfunction (e.g., impaction or tympany) represented only a small number of horses in which typhlotomy was required (*n* = 3). Enterotomy has previously been identified as a risk factor for SSI following equine laparotomy,^34^ but this variable was not significantly associated with SSI in the present study. Whilst the numbers of horses in which typhlotomy was performed was relatively small in both groups, its location close to the midline incision during surgery, potentially increasing risk of bacterial contamination of the incision, or anatomic proximity of the apex of the caecum to a standard midline incision in the standing horse could be plausible factors to explain this finding. Typhlotomy has not been previously reported as a risk factor for SSI following equine laparotomy and warrants inclusion as a variable measured in future similar studies.

Initially, we planned to assess and record dislodgement of the dressings and degree of incisional coverage immediately following anaesthetic recovery and to record digital images of the incisions at bandage changes for truly blinded assessment during hospitalisation. However, it became apparent that this was impractical due to variation in available resources (predominantly personnel time outside routine clinic working hours) and was therefore discontinued. These data were recorded for a total 112 horses (Table 2) and were non‐significant when tested for association with SSI. The proportion of horses in which the primary layer remained intact (i.e., no exposure of the incision) once the horse stood after anaesthesia was higher in the intervention (stent) group (65.5% vs. 50%). Due to the small numbers of horses in each group, we would caution over‐interpretation of these findings. Any differences in these factors between the intervention and control groups in the present study ultimately had no effect on risk of SSI. However, incisional exposure and timing of this during anaesthetic recovery would ideally be recorded in future similar studies, and potentially assessed in a planned subgroup analysis. This requires sufficient resources, particularly personnel available to reliably record such data in a busy hospital setting. It is also possible that there was no difference between groups due to the fact that SSI is multifactorial,^35^ involves various host‐pathogen interactions^36^ and may be more complex in aetiology than simply being a result of physical bacterial contamination of the surgical incision during or immediately following surgery.^37^, ^38^, ^39^

Horse owners and treating veterinary surgeons were unaware of allocation group and incisional assessment following hospital discharge was truly blinded. Potential for detection bias due to outcome assessors being aware of which intervention the patient received^40^ was minimised as much as practicably possible. By deliberately not recording randomisation group on the patient notes, allocated dressing removal being performed immediately following anaesthetic recovery and standardised for both groups thereafter and assessments being conducted by multiple different personnel, assessors were unlikely to be aware of allocation group.

Other limitations of the present study include the fact that this was performed in a single hospital population and therefore the results may not reflect findings from other hospital populations. Multicentre studies are optimal for this reason and are also advantageous in accelerating recruitment of eligible patients. However, these require sufficient resources in terms of personnel and time, frequently limited by funding available and perceptions of funders about the relative importance of SSI following emergency equine laparotomy. The necessity for large scale, rigorous and appropriately randomised trials to fully assess surgical interventions and true efficacy on prevention of SSI in people following laparotomy has been recognised^41^ and is equally relevant to equine emergency laparotomy. Head and tail rope assisted anaesthetic recovery (versus previous hospital protocol for routine free recovery) was introduced part‐way through the study (Summer 2016). Whilst the method of anaesthetic recovery was not recorded as per the trial protocol, published results from our hospital (incorporating all horses in the present study)^22^ demonstrated a significant association between assisted recovery and improved quality of recovery (reduced anaesthetic recovery score). We therefore consider evaluation of anaesthetic recovery score to be valid for the purposes of this study.

This RCT has shown that use of a sutured‐on‐stent compared with a standard adhesive textile dressing as one component of a 3‐layer abdominal bandage for anaesthetic recovery had no effect on SSI following emergency equine laparotomy. Further well‐designed, and ideally individually sufficiently powered, RCT that use standardised outcome measures and appropriate statistical techniques are required to investigate other ways to reduce the risk of this important complication following emergency equine laparotomy. We hope that this study will provide a stimulus for additional randomised, controlled studies to be undertaken in this and other areas of veterinary clinical practice where limited, high‐quality evidence currently exists. Large scale, multicentre studies that are suitably designed and conducted would enable the effectiveness of other potential interventions to reduce the rate of SSI following equine laparotomy to be evaluated rigorously and more quickly but require suitable resources and improved availability of funding.

## FUNDING INFORMATION

The stent bandages for this study were supplied by the manufacturer (Kruuse).

## CONFLICT OF INTEREST STATEMENT

The authors declare no conflicts of interest. The manufacturer who supplied the stent dressings had no input into the design, analysis or reporting of the study.

## AUTHOR CONTRIBUTIONS

**Cajsa M. Isgren:** Conceptualization; investigation; methodology; writing – original draft; writing – review and editing; data curation; project administration. **Gina L. Pinchbeck:** Conceptualization; methodology; writing – original draft; supervision; writing – review and editing; formal analysis. **Shebl E. Salem:** Writing – original draft; formal analysis; writing – review and editing. **Michelle** **J. Hann:** Investigation; writing – original draft; data curation. **Neil B. Townsend:** Conceptualization; investigation; writing – original draft; supervision. **Matthew D. Cullen:** Investigation; writing – review and editing. **Debra C. Archer:** Conceptualization; methodology; writing – original draft; formal analysis; supervision; writing – review and editing; data curation.

## DATA INTEGRITY STATEMENT

Debra C. Archer had full access to all the data in the study and takes responsibility for the integrity of the data and the accuracy of the data analysis.

## ETHICAL ANIMAL RESEARCH

Ethical approval for the study was granted by the University of Liverpool Veterinary Research Ethic Committee (VREC172).

## INFORMED CONSENT

Owners gave informed consent for their horses' inclusion in the study.

## PEER REVIEW

The peer review history for this article is available at https://www.webofscience.com/api/gateway/wos/peer-review/10.1111/evj.14482.

## Supporting information


**Data S1:** Methods S1: Details of incisional protection used during the trial.

**Data S2:** Protocol S1: Sutured‐on‐stent dressing randomised controlled trial.

**Data S3:** Questionnaire S1: Telephone questionnaire for horse owners.

**Table S1:** Summary of the 109 bacterial isolates cultured from 65 horses that developed a surgical site infection (SSI) during hospitalisation.

## Data Availability

The data that support findings of this study are available from the corresponding author upon reasonable request: Open sharing exemption granted by the editor due to lack of provision in the owner informed consent process.
