# Supplementary material for: Evaluation of a stent dressing and abdominal bandage on surgical site infection following emergency equine laparotomy: A randomised controlled trial
Source: Equine Vet J. 2025 Feb 19;57(6):1466–77. doi: 10.1111/evj.14482 (PMC12508274; doi:10.1111/evj.14482)
Supplement: Supplementary file 1 — Data S1: Methods S1: Details of incisional protection used during the trial. [file EVJ-57-1466-s003.pdf]

Equine Veterinary Journal BEVA

METHODS S1: FURTHER DETAILS OF INCISIONAL PROTECTION USED IN THE STUDY

Intervention (sutured-on stent) group

- Kruuse Equine Stent bandage 32 x 15 cm
- Sterile non-adhesive and absorbent wound dressing (no antimicrobial agents)
- 6 x sutures of 4 metric polypropylene placed via reinforced holes to secure stent to skin
- Skin surrounding site dried using sterile swabs
- Kruuse adhesive spray for sterile cover (150ml) applied around site
- Opsite Incise drape applied over stent dressing

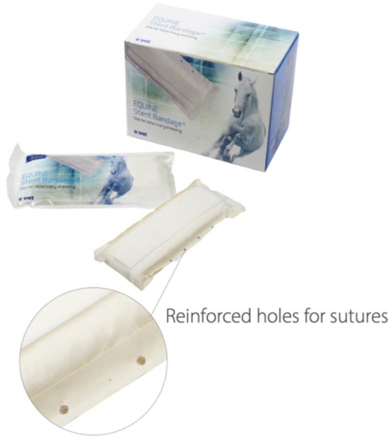

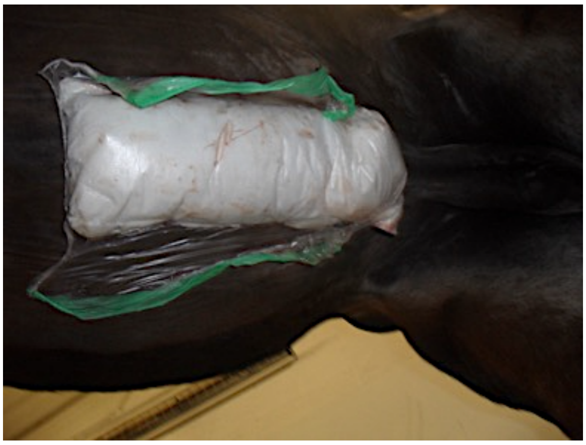

Figure S1.1. Sutured-on stent dressing from manufacturer (now discontinued) shown above; Left. Image of the stent and adhesive drape in place following anaesthetic recovery, after removal of the abdominal bandage

Control (standard adhesive textile dressing) group

- Primapore (Smith & Nephew) 20 x 10 cm non-woven adhesive wound dressing
    - Central non-adhesive component of dressing 156mm x 53mm
    - Adhesive component of dressing 25mm width surrounding the central component
- One or two dressings applied according to incision length to ensure full coverage of incision
- Skin surrounding dressing dried using sterile swabs
- Kruuse adhesive spray for sterile cover (150ml) applied around site
- Opsite Incise drape applied over stent dressing

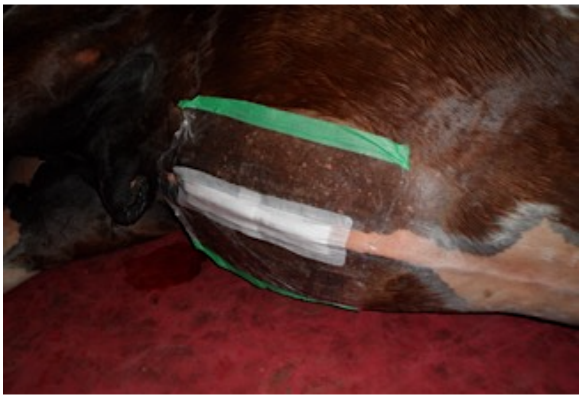

Figure S1.2. Adhesive textile dressing and Opsite incise drape shown in place covering a laparotomy incision in a horse that was not included in the trial. The fabric abdominal bandage used in the trial to cover these dressings is not shown for illustrative purposes.

Elasticated fabric bandage for anaesthetic recovery – intervention and control groups

- Vet Extras - Ortho horse Recovery bandaging (https://www.vetextras.com/products/ortho-horse/recovery-bandaging/)
- 3-dimensional elastic wrap which ends in a wide foam part covered in velour adhesive to Trihook adhesive strip. Interior pocket lined with a monofilm pad 1 cm thick which acts as an air cushion covering the lower abdomen.

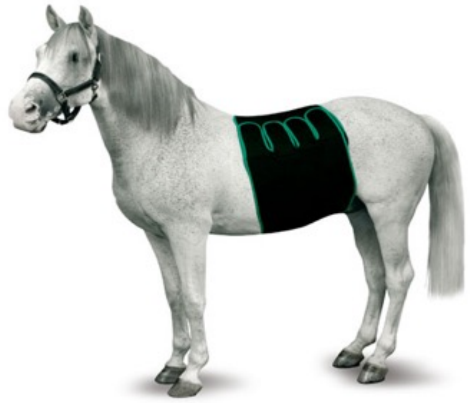

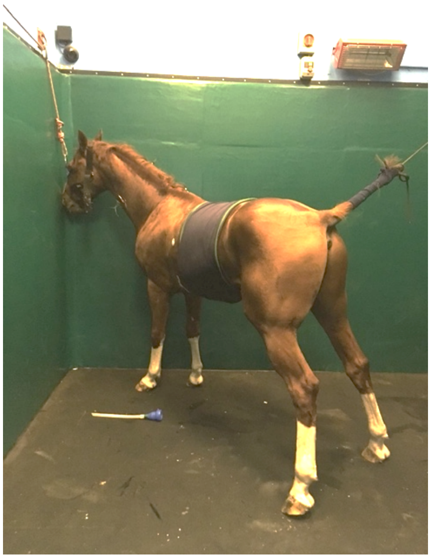

Figure S1.3. Above. Manufacturers image of the recovery bandage; Bottom. Image of recovery bandage showing the interior pocket (white). Left. Image of horse standing immediately following a rope assisted recovery with the three layer method of abdominal protection in place.

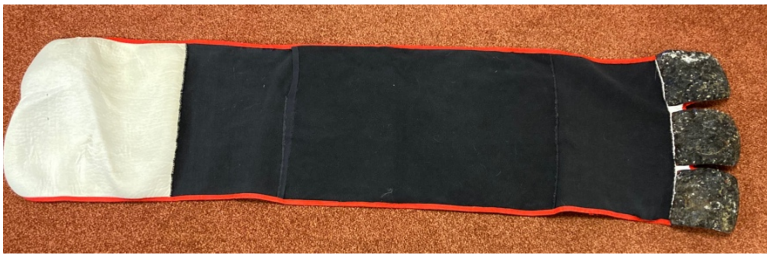

Abdominal bandage placed following anaesthetic recovery and removal of the stent (intervention) or textile adhesive dressing (control)

- All 3 layers of abdominal protection for recovery removed (intervention and control groups)
- Incision managed in a sterile fashion during dressing changes
- Incision covered with a Melolin (Smith & Nephew) sterile dressing
- Kruuse Vet Flex (15cm x 15m) elasticated cohesive bandage applied around the abdomen
- Secured in place cranially +/- caudally with an elastic adhesive bandage (Tensoplast, BSN medical)

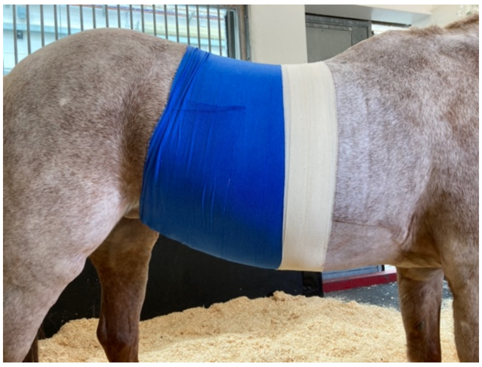

Figure S1.4. Abdominal bandage shown in place.
