# Supplementary material for: Evaluation of a stent dressing and abdominal bandage on surgical site infection following emergency equine laparotomy: A randomised controlled trial
Source: Equine Vet J. 2025 Feb 19;57(6):1466–77. doi: 10.1111/evj.14482 (PMC12508274; doi:10.1111/evj.14482)
Supplement: Supplementary file 2 — Data S2: Protocol S1: Sutured‐on‐stent dressing randomised controlled trial. [file EVJ-57-1466-s004.pdf]

Equine Veterinary Journal BEVA

Protocol S1:

Protocol: Sutured-on-stent dressing Randomised Controlled Trial

Reviewed by CMI, NBT, DCA, GP (October 2013)

Title

Does a sutured-on-stent dressing for incisional protection during anaesthetic recovery reduce the incidence of surgical site infection following exploratory laparotomy compared to a standard textile adhesive dressing?

Introduction

Background and rationale:

Surgical site infection following midline laparotomy is an important complication following laparotomy in horses with rates of 16-26.9% reported (1-4). Infection results in increased hospital stay, increased costs, delayed incisional healing and increased risk of incisional hernia formation requiring potential surgical repair. At the Philip Leverhulme Equine Hospital midline laparotomy incisions are routinely covered with a Primapore dressing followed by an adhesive sheet for recovery following anaesthesia. The dressing often comes off during recovery leaving the surgical site open to contamination. A recent retrospective study using stents to protect midline laparotomy incisions reported reduced infection rates of 2.7% compared to 21.8% without stents for recovery (4). However, this study had a number of biases and other limitations due to its retrospective nature.

Objectives:

To perform a randomised controlled trial to determine if use of a sutured-on-stent for anaesthetic recovery reduces the proportion of horses that develop a surgical site infection (SSI) following laparotomy compared to a standard textile adhesive dressing.

Trial design:

Parallel group randomised controlled trial

Methods - Participants, Interventions and outcomes

Study setting:

Horses undergoing exploratory laparotomy at the Philip Leverhulme Equine Hospital for treatment of colic (acute abdominal pain).

Eligibility criteria:

Horse undergoing exploratory laparotomy from November 2013 onwards with owner informed consent.

Inclusion criteria

- All horses (defined as a horse, pony or donkey) > 1 year of age with owners that provide informed consent for trial inclusion
- Horses in which surgery is completed and the horse walks out of the recovery box. Recovery from general anaesthesia is defined as the horse walking out of the anaesthetic recovery box.

Exclusion criteria

- Equine Grass Sickness confirmed histologically following ileal biopsy

Intervention:

Horses will be randomly assigned to one of two groups for protection of the skin incision just prior to incisional closure. Incisional closure will be performed in normal fashion as per surgeon preference.

1. Stent group - a commercial stent (Kruuse) will be placed over the incision and will be secured in place using six interrupted sutures. An adhesive dressing will be applied over the stent and a textile bellyband placed as the horse is transferred to the recovery box.
2. Control group - a Primapore dressing will be applied to the incision followed by an adhesive dressing and a textile bellyband placed as the horse is transferred to the recovery box.

When the horse is moved out of the recovery box dressings in both groups will be removed and will be replaced by a sterile Melonin dressing followed by an adhesive abdominal bandage. Both groups will be managed in the same way regarding perioperative care based on standard hospital practice and based on clinician preference.

Outcomes:

Primary outcome: the proportion of horses that develop a SSI.

SSI is defined as either positive culture from an incisional discharge, discharge of any purulent material or persistent serosanguinous discharge for more than 48 hours. Mild serous discharge <24 hours post operatively will be considered a normal finding. Dressing changes will be performed every 48 hours unless required to be performed sooner. At each dressing change the surgical site will be assessed by the clinical team, graded as per visual guides and if any discharge is noted a bacteriology swab will be submitted for culture and sensitivity testing.

Participant timeline:

Data will be collected during hospitalization for all horses. Follow up after hospital discharge will be performed via a standardized questionnaire and will be performed at two points; firstly by the referring vet at suture removal and in the first 3 months postoperatively.

Sample Size:
Retrospective data indicate that the current prevalence of post-operative SSI following exploratory laparotomy in the hospital is approximately 20%. We hypothesise that use of a sutured-on-stent will reduce the prevalence of infection by 50% or more which we consider clinically relevant. With 80% power at 5 % significance with 20% prevalence of SSI in the control group and 10% prevalence of SSI in the stent group we require 197 horses in each group i.e. 394 in total.

Methods – Assignment of interventions

Sequence generation:
A random number generator (Excel) will be used to generate a randomised allocation sequence allowing unequal group sizes (allocation ratio 1:1).

Allocation concealment:
A box containing sequentially numbered, opaque, sealed envelopes will be used to randomly assign cases to one of two groups. Inside each sealed envelope will be a paper sheet assigning the horse to either the Stent or Control group for method of incisional protection during anaesthetic recovery. The next envelope in the sequence will be opened once patient eligibility has been confirmed and allocation recorded.

Blinding:
Blinding will be performed by photographs being taken in a standard fashion at each dressing change for each horse. These will be reviewed independently during data analysis.

Methods – data collection, management and analysis

Data collection methods:

Data will be collected using standard hospital forms and will include the following specific data recorded for both groups.

Preoperative data

- Age (years)
- Sex (gelding, stallion, mare)
- Breed (various)
- Weight (kg)
- Date of surgery
- Day of the week of surgery (Mondays to Sunday)
- Time of induction of anaesthesia
- Month of surgery (January- December)
- Heart rate on arrival (beats per minute)
- Packed cell volume on arrival (%)

- Systemic total protein on arrival (g/L)
- Systemic lactate on arrival (mmol/L)
- Peritoneal total protein on arrival (g/L)
- Peritoneal lactate on arrival (g/L)
- Coat condition on arrival (clean clipped/clean unclipped/moderate/filthy)

Perioperative data

- Primary surgeon (various)
- Primary surgical lesion (various)
- Decompression of SI only (Y/N)
- SI resection (Y/N)
- Length of resection (feet)
- SI resection – EEJJA (Y/N)
- SI resection – EEJIA (Y/N)
- SI resection – SSJCA (Y/N)
- LC resection (Y/N)
- LC reposition (Y/N)
- LC enterotomy (Y/N)
- SC enterotomy (Y/N)
- SC resection (Y/N)
- Number of layers used to close the midline (2/3)
- Suture material for linea alba (vicryl / other)
- Suture material for subcutaneous layer (vicryl/ other/none)
- Suture material for skin (prolene/ vicryl / staples/other)
- Suture pattern for linea alba (simple continuous/ halves of simple continuous/centre cruciate followed by halves of simple continuous/ other)
- Suture pattern for subcutaneous layer (subcutaneous /other)
- Suture pattern for skin (Ford interlocking / simple continuous/ none i.e. staple)
- Duration of general anaesthesia (minutes) (defined as from time of induction to disconnection from breathing circuit)
- Duration of surgery (minutes) (defined as time from first incision to last suture placement)
- Depth of thickest adipose tissue of midline prior to closure as measured with sterile ruler (cm)
- Length of incision prior to closure as measured with sterile ruler (cm)
- Recovery dressing (primapore followed by opsite adhesive & textile abdominal bandage) or sutured sterile stent followed by opsite adhesive & textile abdominal bandage
- Recovery score following general anaesthesia as per Guelph chart (1-6)
- Was the incision covered for the duration of recovery (Y/N)

    - Type antimicrobial administered prior to surgery (sodium penicillin and gentamicin/ procaine penicillin and gentamicin/ other)

Follow up data

- HR @24h (beats per minute)
- Temperature @24h (C)
- PCV@24h (%)
- TP@24h (g/L)
- HR@ 48h (beats per minute)
- Temperature @48 h @ (C)
- PCV @48h (%)
- TP @48h (g/L)
- Colic signs post operatively (Y/N)
- Post operative pyrexia (defined as rectal temperature of >38.5 C on at least one occasion)(Y/N)
- POI signs following surgery (Y/N) (defined as functional complication of surgery in horses where >2L reflux was gained via nasogastric intubation on 2 or more occasions)
- Lidocaine continuous rate infusion given post operatively? (Y/N)
- Peritonitis (Y/N) (defined as two or more parameter including peritoneal white blood cell of >60 x 10 9 cell/L, peritoneal neutrophil count > 70%, peritoneal total protein > 60 g/L, increased peritoneal fluid on abdominal ultrasonography)
- Type of antimicrobial treatment (penicillin only/ penicillin and gentamicin/ other)
- Duration of antimicrobial treatment (days)
- Duration of hospital stay following surgery (days)
- SSI present (defined as any purulent or persistent serosanguinous drainage for more than 48 hours or with a positive culture) (Y/N)
- Pain on palpation @ 1st dressing change (none/mild/mod/severe)
- Wound discharge @ 1st dressing change (Yes/No)
- If discharge type of discharge (serosanguinous/ purulent/ haemorrhagic/other)
- Oedema @ 2nd dressing change (none/mild/mod/severe)
- Pain on palpation @ 2nd dressing change (none/mild/mod/severe)
- Wound discharge @ 2nd dressing change (Yes/No)
- If discharge type of discharge (serosanguinous/ purulent/ haemorrhagic/other)
- Oedema @ 3rd and consecutive dressing change (none/mild/mod/severe)
- Pain on palpation @ 3rd and consecutive dressing change (none/mild/mod/severe)
- Wound discharge @ 3rd and consecutive dressing change (Yes/No)
- If discharge type of discharge (serosanguinous/ purulent/ haemorrhagic/other)
- Presence of positive culture of discharge (Y/N)

- Bacteria and sensitivity results (descriptive)
- Pattern of sensitivity (descriptive)
- Development of hernia (Y/N)
- Repeat laparotomy (Y/N)

SSI reporting:
Swabs will be submitted for general aerobic and anaerobic bacterial culture to the microbiology laboratory at Leahurst Campus. Bacterial culture will consist of direct plating onto 5% sheep blood agar (Oxoid, UK) and the plates will be incubated aerobically and anaerobically for 2-7 days. Microorganisms isolated from positive cultures will be identified using API kits (Biomerieux, France) and also with the GNID Sensititre Identification plates (TREK Diagnostic Systems, West Sussex, UK).

Statistical methods:

Initial analysis will include a baseline comparison of group characteristics to check the adequacy of randomisation.

Data will be analysed according to intention to treat principles (ITT). Baseline data for each group will be assessed and continuous variables reported as means and standard deviation (Normally distributed data) or as median and interquartile range (IQR) for non-Normally distributed data. Baseline categorical data will be assessed and reported as numbers and proportions. The primary outcome (days to SSI) will be analysed using time-to-event analysis. Horses will be censored at the point of death / euthanasia or at time of loss to follow up. Time to SSI for each group will be estimated using Kaplan Meier plots and a Cox proportional hazard model.

Research ethics approval

Ethical approval will be submitted to the Veterinary research ethics committee and approval obtained prior to trial commencement

References

1. Coomer, R. P. C., Mair, T. S., Edwards, G. B. and Proudman, C. J. (2007), Do subcutaneous sutures increase risk of laparotomy wound suppuration?. Equine Veterinary Journal, 39: 396–399

2. Mair, T. S. and Smith, L. J. (2005), Survival and complication rates in 300 horses undergoing surgical treatment of colic. Part 2: Short-term complications.

Equine Veterinary Journal, 37: 303– 309

3. Proudman, C. J., Smith, J. E., Edwards, G. B. and French, N. P. (2002), Long-term survival of equine surgical colic cases. Part 1: Patterns of mortality and morbidity. Equine Veterinary Journal, 34: 432–437

4. Tnibar, A., Grubbe Lin, K., Thurøe Nielsen, K., Christophersen, M. T., Lindegaard, C., Martinussen, T. and Ekstrøm, C. T. (2013), Effect of a stent bandage on the likelihood of incisional infection following exploratory coeliotomy for colic in horses: A comparative retrospective study. Equine Veterinary Journal, 45: 564–569
