# Supplementary material for: Evaluation of a stent dressing and abdominal bandage on surgical site infection following emergency equine laparotomy: A randomised controlled trial
Source: Equine Vet J. 2025 Feb 19;57(6):1466–77. doi: 10.1111/evj.14482 (PMC12508274; doi:10.1111/evj.14482)
Supplement: Supplementary file 3 — Data S3: Questionnaire S1: Telephone questionnaire for horse owners. [file EVJ-57-1466-s002.pdf]

Equine Veterinary Journal BEVA

Questionnaire S1: Telephone questionnaire for horse owners

Number in study

Phone Number (1):

Phone Number (2):

Sequential swabbing Colic follow-up Questionnaire

Name of owner: Name of Horse:

Date of surgery: Date of follow-up:

Surgical Lesion:

Introductory statement: I am telephoning with regards to XX , your horse that underwent colic surgery at Leahurst. We are following horses after surgery to monitor their progress at home. Your participation is voluntary and may withdraw consent at any time. All information is confidential.

Would you be prepared to answer a short questionnaire? **Yes / No**

Questions regarding survival

1. Do you still own the horse? **Yes / No**
2. Is the horse still alive? **Yes / No**
3. If No: was euthanasia related to the colic /wound? **Yes / No**
4. Was euthanasia unrelated to colic /wound? **Yes / No**
5. Please specify the reason for euthanasia ________________________________
6. Date of euthanasia ___________________________

Questions regarding post-operative care following discharge:

Colic Episodes

1. Did the horse experience any further colic episodes? **Yes / No**
   -Date(s)?
2. Number of colic episodes in total?
3. Did the vet attend? **Yes / No**

Cont. on next page

Incisional Problem

4. Did the horse develop any problems with the incision? **Yes / No**
   - Drainage **Yes / No**
     - Serous / purulent
   - Oedema **Yes / No**
   - Hernia **Yes / No**
   - Did the vet attend? **Yes / No**
   - Was a sample for bacteriological culture obtained? **Yes / No**

     If so, can you remember what bacteria was grown?.........

Diarrhoea

5. Did the horse experience loose droppings? **Yes / No**

   - Did the vet attend? **Yes / No**

6. Did the horse experience weight loss requiring vet intervention? **Yes / No**

Questions regarding return to athletic function

7. Did the horse return to work? **Yes / No / NA**
8. What level of work was the horse in prior to colic surgery?
   - No work
   - Leisure horse (light hacking/ schooling)
   - Racing
   - Affiliated competition horse: BE/ BSJA/ BD/ other
   - Low level/ unaffiliated competition horse
   - Other (please specify)______________________

5. To what level of work has the horse returned to?
   - No work
   - Lower than previously
     - Is there a reason for lower level work?
   - Same as previously
   - Higher than previously

6. If competition horse, has it returned to competing? **Yes / No / NA**
