# Supplementary material for: Evaluation of a stent dressing and abdominal bandage on surgical site infection following emergency equine laparotomy: A randomised controlled trial
Source: Equine Vet J. 2025 Feb 19;57(6):1466–77. doi: 10.1111/evj.14482 (PMC12508274; doi:10.1111/evj.14482)
Supplement: Supplementary file 4 — Table S1: Summary of the 109 bacterial isolates cultured from 65 horses that developed a surgical site infection (SSI) during hospitalisation. [file EVJ-57-1466-s001.pdf]

Equine Veterinary Journal BEVA

Table S1: Summary of the 109 bacterial isolates cultured from 65 horses that developed a surgical site infection (SSI) during hospitalisation.

Bacterial culture was performed by direct plating onto 5% sheep blood agar. Plates were incubated aerobically and anaerobically for 2–7 days at 37.0°C. Results were reported as pure or mixed growth with all isolates identified at species level using API kits[f] and GNID and GPID Sensititre Identification plates[g]. Oxacillin or 3rd generation cephalosporin resistant isolates were further investigated for methicillin-resistant *Staphylococcus aureus* (MRSA) and extended-spectrum β-lactamase (ESBL) producing *Enterobacteriaceae*.

Bacterial isolate	Intervention N (%)	Control N (%)	Total
Gram-negative	28 (49.1)	29 (50.9)	57
Gram-positive	26 (50.0)	26 (50.0)	52
Staphylococcus spp.	15 (44.1)	19 (55.9)	34
of which Staphylococcus aureus	9 (50.0)	9 (50.0)	18
of which MRSA	7 (63.6)	3 (27.3)	11
Escherichia coli	14 (48.3)	15(51.7)	29
Enterococcus spp.	9 (64.3)	5 (35.7)	14
Pseudomonas spp.	3 (50.0)	3 (50.0)	6
Klebsiella pneumoniae.	2 (40.0)	3 (6.0)	5
Enterobacter spp.	2 (50.0)	2 (50.0)	4
Morganella spp.	2 (50.0)	2 (50.0)	4
Acinetobacter baumanii	1 (50.0)	1 (50.0)	2
β-haemolytic Streptococcus spp.	1 (50.0)	1 (50.0)	2
Other	5 (55.6)	4 (44.4)	9

MRSA = methicillin-resistant Staphylococcus aureus; The category "other" was used for bacteria present in low numbers or those considered not to be of clinical significance.
